# California Wines

**Published:** 1867-10

**Authors:** 


### *California Wines.*

European prestige sustains the sale of vile dilutions of whiskey known as wines. The "pure" or "simon-pure" article is, not unfrequently, as bad or worse. There is little doubt that California is one of the best (if not the best) grape producing countries known. Yet California wines have been in comparative disrepute, because of the sale of common rhubarb wine (*et alii*) under the same denomination. Nevertheless, pure and delightful wines are produced in the modern El Dorado. The advantage of using pure wines in therapeutics, rather than fiery distillates from corn, rye, or molasses, will readily occur to the professional mind. But to get them, *hoc opus*. From the best information we can obtain, we believe they can be procured in their pristine purity, delicious as the nectar of the gods, and with phases of taste and aroma as diverse as the tints of the rainbow, by applying to the El Dorado Wine Company, Chas. P. Jackson, Esq., General Sup't, 117 Randolph Street, in this city. There can be no doubt of the origin and perfection of the articles which they furnish.

